# Global landscape assessment of screening technologies for medicine quality assurance: stakeholder perceptions and practices from ten countries

**DOI:** 10.1186/s12992-018-0360-y

**Published:** 2018-04-25

**Authors:** Lukas Roth, Ameena Nalim, Beth Turesson, Laura Krech

**Affiliations:** Global Public Health Division, United States Pharmacopeial Convention, 12601 Twinbrook Parkway, Rockville, Maryland USA

**Keywords:** Pharmaceutical systems, Medicine quality screening technologies, Substandard and falsified medicines

## Abstract

**Background:**

The spread of substandard and falsified (SF) medical products constitutes a growing global public health concern. Some countries use portable, handheld screening technologies (STs) in the field to accelerate detection of SF medicines and reduce the number of medicine samples that undergo costly and time-consuming confirmatory analysis.

**Methods:**

A multi-country, multi-stakeholder landscape assessment utilizing qualitative methodology was used to examine practices and perceptions related to the use of STs. Qualitative interview guides were designed using the results of a literature review and comprised of open-ended questions with the study participants, who were from national medicine regulatory authorities, pharmaceutical manufacturers, pharmacies, and distributors. Ten geographically and economically diverse countries were selected: Argentina, China, Egypt, India, Jordan, Mexico, Nigeria, Philippines, the United States, and Zimbabwe. Of the completed 53 interviews, 32 were in-person, 16 were phone interviews, and 5 were via written questionnaires.

**Results:**

Data analysis shows a wide variation in understanding and usage of STs in different sectors. Virtually all of the study participants indicated a lack of objective, accessible information on STs to advise them on what technologies would be beneficial for their needs. Study participants also described their ideal capabilities of the next generation of STs, including shareable spectral libraries, lower acquisition costs, lesser training requirements, and in-country maintenance and technical support.

**Conclusion:**

The results and recommendations presented in this article can be used to help regulators communicate and justify their needs to acquire and invest in new STs. There is a need for additional standardized, trustworthy and scientifically sound evaluations of STs, and to support regulators to effectively deploy the most promising technologies. ST manufacturers can take into account some of the limitations of the technologies the interviewees identified in this article, such as a dearth of technologies, which provide quantitative information about the active ingredient, and take steps to address them to better serve their customers. These results and recommendations, can catalyze research and actionable interventions into the development, review, application, and use of STs.

**Electronic supplementary material:**

The online version of this article (10.1186/s12992-018-0360-y) contains supplementary material, which is available to authorized users.

## Background

The spread of substandard and falsified (SF) medical products continues to be a growing global concern [1–3]. Their prevalence in the public, private, and informal market sectors threatens global public health by jeopardizing patient safety, diminishing confidence in health systems, increasing treatment failure, wasting valuable resources, and contributing to the development of drug resistance [1, 4–6]. Although SF medicines negatively impact public health in both developed and developing nations, available data unequivocally demonstrate that developing countries have greater numbers of poor quality medicines circulating in their markets and, as a consequence, suffer greater health burdens [1, 2, 7, 8].

Accurate global estimates of the prevalence of poor quality medicines do not exist [1, 4, 9, 10]. However, a reasonable prevalence estimate for falsified medicines in developing countries ranges from 10% to 30% [11, 12]. The literature suggests that the global market for falsification of medicines, at $431 billion USD per year, may be on par with that of illicit drugs, at $435 billion USD per year [13, 14]. The World Health Organization (WHO) is in the process of completing a study on the public health and socioeconomic impact of SF medical products based on published, reliable surveys conducted over the past 10 years. This study will provide a benchmark against which to calibrate future responses to SF medicines.

Over the last 5 years, research in screening technologies (STs) has expanded to more than 20 unique, portable technologies available to address poor quality medicines. Some STs are commercially available while others are still in the process of development or field testing [6, 7, 15–17]. The screening analysis methods currently used in countries include physical, visual, chemical, and microbiological analyses. By confirming ink color, language, spelling, shape, size, and other variable data, visual and physical inspection of the finished pharmaceutical product and its packaging can determine a suspected poor quality product due to wrong or tampered packaging [18]. Tablets, capsules, and liquids can also be examined for imprint, color, and odor irregularities, as well as visible contamination. Alternate light sources can also be employed. For example, the U.S. Food and Drug Administration Counterfeit Detection Device Version 3 uses ultraviolet and infrared light to identify falsified products [19]. Chemical analysis can identify medicines with little or no active ingredient or incorrect active pharmaceutical ingredient by using colorimetric tests, thin-layer chromatography, or various forms of spectroscopy. Finally, microbiological analysis can demonstrate the potency of antibiotics and sterility of injectable drug products.

A comprehensive article in 2014 by Kovacs et al., details the available field and laboratory STs for identifying poor quality medicines, including each technology’s need for electricity, sample preparation, reagents, portability, level of training required, and speed of analysis [15]. In addition, all STs were categorized by cost: $10,000 USD; $10,000 to $100,000 USD; and greater than $100,000 USD. The paper identified two key issues when examining STs: 1) performance data was not always available for each technology in use or STs under development; and 2) a gold standard was lacking as a comparator for all technologies available to detect SF medicines. Lastly, a WHO Collaborating Center survey in 39 countries outlined the quality control laboratory techniques (e.g., chromatography, spectrophotometry) used to test for SF medicines, but it did not differentiate between traditional, laboratory-based techniques, and STs used in the field [20].

Developing countries urgently need inexpensive, easy-to-use, portable, and rapid methods to detect poor quality medicines and diagnostics throughout the supply chain [21–25]. STs are not meant to obviate the need for a functional Official Medicines Control Laboratory (OMCL), but they should be an integral part of a country’s quality assurance toolkit, particularly in low and low middle-income countries where poor quality medicine prevalence is higher and the number of highly trained lab technicians is lower.

The aim of screening is to reduce the number of samples an OMCL must test, which subsequently reduces the burden on the laboratory and its limited resources [21]. Screening methods can identify suspect products, but they cannot replace confirmatory quality control testing required by each country’s legal framework [20]. Making these technologies more accessible will help control the proliferation of SF medicines, protect consumers, and generate accurate estimates for the prevalence of poor quality medicine [6].

In summary, there is a technology gap in the development of affordable, easy to use, and precise STs. There is also an information gap of standards, data, and objective evaluations needed to compare and contrast STs so that countries can decide which are most appropriate for their needs. The purpose of this study is to carry out a global landscape assessment of the benefits and limitations of STs and to more accurately ascertain current practices and country needs.

## Methods

This research study was composed primarily of a literature review and a qualitative research component. This article focuses on STs that are available for finished pharmaceutical products. It does not cover authentication technologies (e.g., holograms, fluorescent inks, barcodes, chemical taggants) that enable authentication of a medical product but often do not assess its key quality attributes (e.g., active pharmaceutical ingredient identity). In the context of this article, the term “medicines” includes branded and generic finished pharmaceutical products for all types of therapeutic indications and dosage forms. Substandard and Falsified medicines, as defined by the WHO Member State Mechanism, includes those that are substandard or out of specification (i.e., authorized medical products that fail to meet either their quality standard or their specifications, or both) and falsified (i.e., medical products that deliberately or fraudulently misrepresent their identity, composition, or source). The term, “poor quality medicines” encompasses all SF medicines. “Track and trace systems” are not considered medicine-quality ST; however, they are useful and efficient tools for fighting the distribution of poor quality products. Examples of track and trace methods include radio frequency identification, traditional and two-dimensional barcodes, microtags, nanoencryption, and mobile verification.

The literature review focused on the problem and scope of poor quality medicines globally and the current practices relating to the application of STs for assuring the quality of medicines and their relative advantages and limitations. The search terms used were “poor quality medicines,” “substandard medicines,” “falsified medicines,” “counterfeit medicines,” “screening technologies,” “detection technologies,” “medicine screening,” “medicine testing,” “counterfeit medicine detection,” “substandard medicine detection,” “falsified drug detection,” “poor quality medicine detection,” and substituting the term “drugs” for medicines for all search terms. PubMed, Web of Science, Google Scholar, Essential-drug list serve, and other available databases, including the WHO’s global digital library, Worldwide Antimalarial Resistance Network’s Antimalarial Quality Literature Surveyor, and the U.S. Agency for International Development’s Development Experience Clearinghouse, were used to search for literature. The literature review focused on articles from 2012 to October 2016. The justification for this time period being that the report, Countering the Problem of Falsified and Substandard Drugs in 2013 captured voluminous citations on the scope and problem of poor quality medicines globally and the article Technologies for Detecting Falsified and Substandard Drugs in Low- and Middle-Income Countries in 2014 provided a comprehensive list of STs that exist [6, 15].

The goal of the qualitative research component was to elicit a wide range of responses from the survey participants to better understand the current landscape. A multi-country, multi-stakeholder landscape assessment utilizing qualitative methodology was used to examine practices and perceptions related to the use of STs. Qualitative interview guides were designed using the results of a literature review and comprised of open-ended questions with the study participants, who were from national medicine regulatory authorities (MRAs), pharmaceutical manufacturers, pharmacies, and distributors. Ten geographically and economically diverse countries were selected: Argentina, China, Egypt, India, Jordan, Mexico, Nigeria, Philippines, the United States, and Zimbabwe. Of the completed 53 interviews, 32 were in-person, 16 were phone interviews, and 5 were via written questionnaires.

### Country selection procedure

**Fig. 1:**
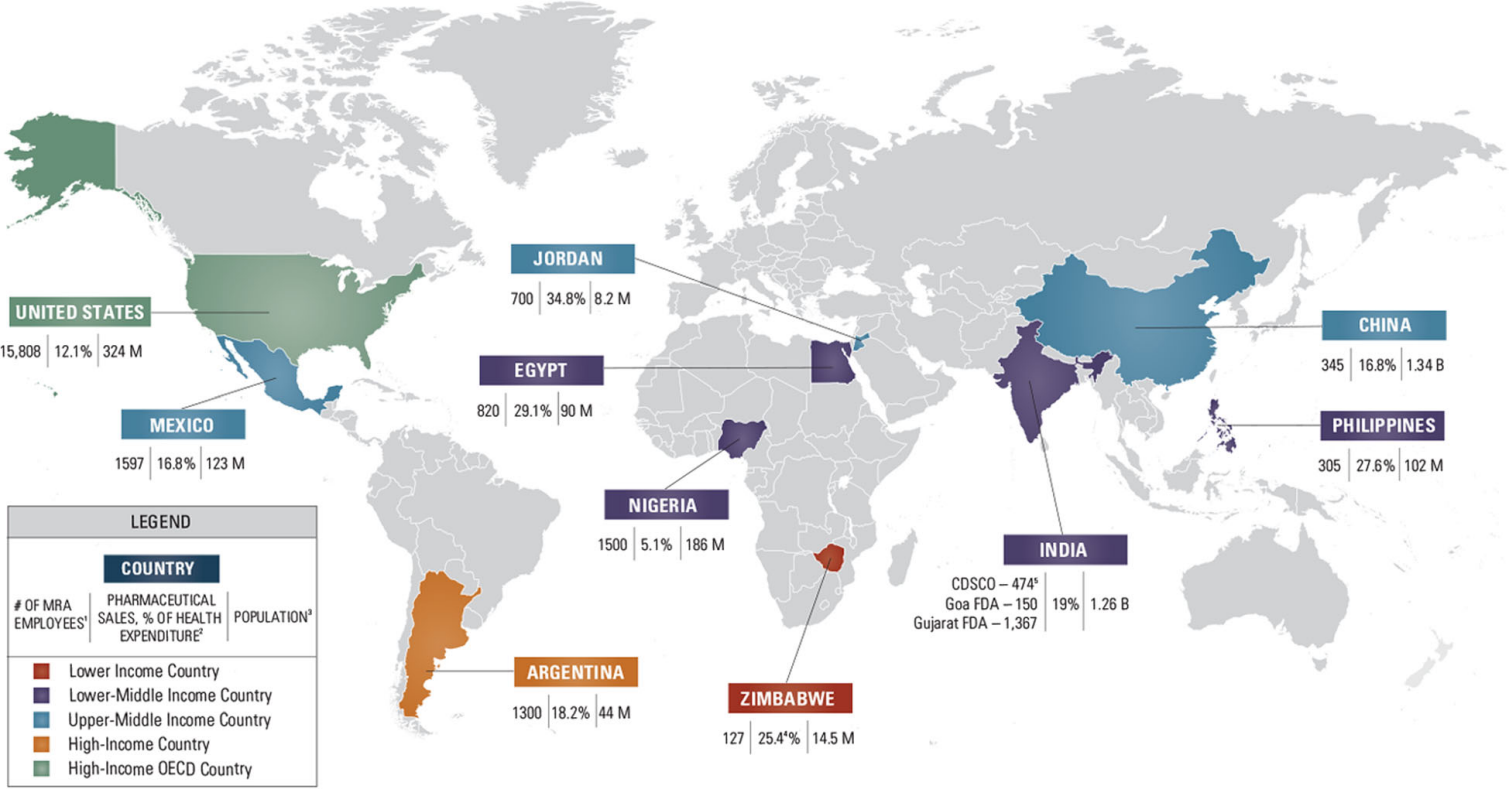
Key Country Selection Criteria Considered — World Bank development classification, number of MRA employees, pharmaceutical sales, and population. ^1^Estimates based on information obtained from interviews and available literature and reflect predominantly numbers associated with Federal level staff. Apart from those provided for the Goa FDA (a state level institution), these numbers do not include provincial, state, city level staff, or contractors. ^2^Business Monitor International (BMI). ^3^United States Census Bureau: International Database [database on the Internet]. Available from: https://www.census.gov/popclock/world. ^4^BMI and WHO Global Health Expenditure Database. ^5^ Babu, G. (2017). Personnel shortage, lack of funding hold back India’s drug regulator. [online] Business-standard.com. Available at: http://www.business-standard.com/article/companies/personnel-shortage-lack-of-funding-hold-back-india-s-drug-regulator-115111100633_1.html [Accessed 13 Oct. 2017]

Country selection criteria are listed in Fig. 1 and Table 1. Local manufacturing capacity, while not explicitly used as criteria for country selection, was considered. For example, Argentina, China, India, Nigeria, and the United States are major pharmaceutical manufacturing countries globally and in their respective regions.

**Table 1:** Key Country Selection Criteria — SF and MRA information

Region	Recent SF Information	MRA Information
Africa	Nigeria	
	▪ One of the countries with the highest reported prevalence of poor quality medicines [10, 35]. ▪ Extensive publicity on poor quality medicines circulating as a result of importation and local manufacture [10, 33]. ▪ Large local pharmaceutical industries, with references citing that poor quality medicines produced locally have been diverted to other countries[36] ▪ Some STs (Minilab™ and TruScan™) have been deployed domestically [37, 38].	▪ The National Agency for Food and Drug Administration and Control (NAFDAC) has recently made efforts to decrease the amount of poor quality medicines in circulation. However, pharmaceutical regulations in many cases are still not implemented and enforced [38, 39].
	Zimbabwe	
	▪ Limited public information available about poor quality medicines in country. ▪ In 2003, WHO examined the quality of selected antimalarials and found quality problems with both chloroquine and sulfadoxine pyrimethamine tablets [40]. ▪ Widespread poverty and stock outs have forced some people to obtain their medicines from unlicensed vendors selling falsified products [41].	▪ Despite economic challenges, the Medicines Control Authority of Zimbabwe (MCAZ) is one of Africa’s regulatory success stories and has provided technical assistance to neighboring countries. The OMCL is WHO prequalified and ISO 17025 accredited [42].
Americas	Argentina	
	▪ Falsification of medicines is cited as a serious, systemic problem in which leaders from various sectors participate: politics, business, and labor [43]. ▪ As part of continuing investigations, over 40 raids were recently conducted into falsified and illegal drugs distributed by organized crime. The police in collaboration with the National Administration of Drugs, Foods and Medical Devices (ANMAT) collected samples of primarily cancer, hemophilia, and HIV/AIDS medicines as well as documentation related to their purchase and distribution [44].	▪ The ANMAT is recognized by the Pan American Health Organization as a National Regulatory Authority of Regional Reference [45]. ▪ Since 1997 Argentina created the National Research Program of Illegitimate Medicines, to determine the magnitude of SF medicines on the market and limit their circulation and public health consequences [46].
	Mexico	
	▪ Extensive local production and import of falsified medicines and instances of poor quality medicines were found at US-Mexico border pharmacies [47]. ▪ Illicit drug cartels are also involved in the distribution chains of falsified medicines [9]. ▪ A recent study analyzed the active pharmaceutical ingredients from local pharmacies of 17 commonly used products, with several units falling outside of U.S. Pharmacopeia standards specifications [48].	▪ Mexico’s Federal Commission for Protection against Sanitary Risks (COFEPRIS) is recognized by the Pan American Health Organization as a National Regulatory Authority of Regional Reference [49]. ▪ Mexico’s OMCL is WHO prequalified [42].
	United States of America	
	▪ Falsified medicines are becoming more prevalent in the United States. Most are “lifestyle” medicines but there are exceptions, notably anti-cancer, anti-depressant and anti-anxiety medicines [3, 50]. ▪ Media reports contain several examples of falsified medicines seized at borders, which were imported illegally [3, 50]. ▪ Locally produced falsified medicines have been discovered, but medicine seizures indicate that the majority of falsified products are either imported or smuggled in across borders [51]. ▪ An estimated 80% of falsified medicines come from overseas [2, 6]	▪ The U.S. Food and Drug Administration (USFDA) regulates over-the-counter and prescription drugs, including biological therapeutics and generic drugs. ▪ USFDA recently established the Office of Pharmaceutical Quality, which is dedicated explicitly and exclusively to product quality.
Eastern Mediterranean	Egypt	
	▪ Media reports claim that falsified medicines are now estimated to make up 30% of the Egyptian market; in 2015, a series of raids found counterfeit medicines worth hundreds of millions of dollars and exposed a criminal network feeding consumers across Middle East [52]. ▪ This problem is tightly linked to pharmacies with multiple branches whose owners hold huge capital and are close to Egyptian decision makers; they are the ones primarily smuggling and selling falsified medicines. ▪ Egypt serves as a transit hub for falsified medicines destined to other countries [53, 54].	▪ The Egyptian Drug Authority has its own website and is the pharmaceutical regulatory body of the Egyptian Ministry of Health. The Authority is composed of three sub-organizations: Central Administration of Pharmaceutical Affairs, the National Organization for Drug Control and Research (NODCAR) and National Organization for Research & Control of Biologicals.
	Jordan	
	▪ In 2008, 431 pharmacies were found in violation of law; 14 were shut down for selling counterfeit medicines and 34 were selling smuggled medicines [55]. ▪ In 2013, the Jordanian Food and Drug Administration (JFDA) seized USD $307,682 of counterfeit medicines and issued warnings to 21 manufacturers over the course of six months [56].	▪ JFDA’s OMCL is in the process of obtaining ISO 17025 accreditation.
Southeast Asia	India	
	▪ Global supplier of generic medicines, although literature exists of local manufacturers producing and exporting substandard and falsified medicines [10, 50]. ▪ Reports claim that some local manufacturers have two-tiered production, making substandard drugs for markets with poor regulatory authorities in Africa [36, 57]. ▪ Large manufacturer, Ranbaxy, recently pled guilty to felony charges for adulterating medicines [58].	▪ Regulatory functions are decentralized; state and national-level institutions are given different powers [10]. ▪ State-level agencies license and monitor drug manufacturing establishments and drug testing laboratories, regulate medicine quality, and approve drug formulations [9]. ▪ Overall, the state regulatory authorities are weak and lack adequate quality testing facilities [59]. ▪ Large personnel and budget shortages at both levels [60].
Western Pacific	Philippines	
	▪ Numerous reports of poor quality medicines, both falsified and substandard [36]. ▪ In 2015, the Philippines Food and Drug Administration (FDA) ordered the seizure of 20 different unregistered medicines [61]. ▪ In 2015, two customs officials were fired after releasing a shipment of counterfeit medicines worth USD $269,000 [62].	• There is a strong public bias against generic medicines, with people spending large amounts to purchase originator products or ‘higher-quality’ branded generics [63]. • The Cheaper Medicines Act was therefore established by FDA to demonstrate the quality of generics [63].
	China	
	▪ Production value of domestic pharmaceutical industry increased from 137.1 billion yuan to 667.9 billion yuan from 1997 to 2007 [64]. ▪ Along with India and Nigeria, China has reports of SF medical products which can be attributed in some cases to substandard local manufacturing and are sometimes exported [10, 64]. ▪ China and India manufacture 70–80% of the active pharmaceutical ingredients for all medicines globally [57, 65].	▪ China has recently been cracking down on poor quality medicines, giving the State FDA additional resources to supervise, implement regulations, and to initiate investigations into and enforce penalties for violations [66, 67]. ▪ Recent reforms have emphasized transparency, strict regulatory standards, and enforcement mechanisms and China requires manufacturers to follow WHO’s Good Manufacturing Practices for pharmaceutical active ingredients and finished products [6, 67].

#### The following factors were used for country selection:

- **Geography:** Countries were divided into regions using the WHO classification (i.e., Africa, Americas, Eastern Mediterranean, South-East Asia, Europe, and Western Pacific). Europe was the only region not included in this study (the Limitations section provides details).
- **World Bank Development Classification:** countries were selected from all classification areas ranging from low-income to high-income Organization for Economic Co-operation and Development countries [26]
- **SF data:** for each potential country, SF data, reports, and articles were examined. After preliminary selection, countries were included if literature indicated local problems with SF medical products.
- **MRA functionality:** pharmaceutical sector analyses were available for many countries and if not, available information on country MRAs was examined. The number of MRA employees was noted in the selection process, as this can be an indicator of an MRA’s budget and capacity to carry out its core functions.
- **Availability and willingness of countries to participate in the study:** countries needed to be available and willing to host the qualitative researcher and arrange interviews with the country MRA, OMCL, manufacturers, and pharmacies or to arrange for the interviews to be done via phone.
- **Pharmaceutical sales as a percentage of health expenditure:** countries where pharmaceutical sales as a percentage of total health expenditure was varied were selected.
- **Country population estimates for 2016:** the number of country inhabitants was considered. The study wanted to examine countries with large populations (over 1 billion) and countries with smaller populations (less than 15 million) [27].

### Interview guide development

Qualitative research methods, such as in-depth interviews, were determined to be the best approach to soliciting current information about STs in use in the selected countries. The in-depth interview guides, including the types of questions to be asked, additional probes, and which personnel should be interviewed, were constructed based on the results of the literature review. Interviews were conducted and participants were asked open-ended questions on the following: the use of STs (i.e., when, where, how, and why) to detect poor quality medicines along the supply chain by their respective organizations; the types of STs in use and reasons for using them; the benefits and limitations of using these STs; and what is needed to better utilize STs and what would be characteristics of the ideal ST.

Based on the literature review, interview guides were developed (see Additional file 1) for the different groups participating in the study (MRA and OMCL staff, pharmaceutical manufacturers, and distributors and pharmacies. All interview guides covered issues around medicine quality issues, the status of STs, and post-marketing surveillance (PMS) activities being carried out in their respective countries.

### Sampling plan

In-depth, semi-structured interviews of key-informants representing government regulators (R), manufacturers (M), and distributors and pharmacies (DP), were carried out in 10 countries from April–September 2016. For each country, interviewees included key staff from the national MRA and quality control laboratory, pharmaceutical manufacturers (private and public, where possible), and distributors and pharmacies (private and public, where possible).

### Data collection

The participants represented various sectors involved in assuring the quality of medicines in their respective countries; participants self-identified as regulators, manufacturers, distributors or pharmacists in each of the 10 countries.

Recruitment of participants was done in multiple ways. With the help of U.S. Pharmacopeia contacts on the ground in each country, the study team contacted persons who agreed to be interviewed. Prior to the interview, participants had been informed of the interviewer’s arrival in country (or in the case of telephone interviews, a specific date and time was agreed upon) and had been briefed on the goals and objectives of the study. In most countries, interviews were carried out until saturation was reached for the issues around the use of STs. Interviews were done in English, with the exception of Mexico and Argentina where the interviews were carried out in Spanish. To maintain reliability, only two researchers carried out the interviews; the lead qualitative researcher carried out interviews for eight countries and a second researcher, who was trained to use the interview guides by the qualitative expert, conducted the Spanish-speaking interviews.

Where in-person interviews were not available, phone interviews were carried out. In a few instances with regulators and manufacturers, interviews were not an option; therefore, written questionnaires in English were sent out to these participants. All in-person interviews, phone interviews, and written questionnaires used the same interview guides specific for each organization.

Interview guides for R, M, and DPs, with open-ended questions to guide the interviews, were prepared prior to data collection. However, some spontaneous generation of questions also took place as the interview progressed and necessitated follow-up or probing questions to solicit details on topics that emerged during the interview.

### Data analysis

Data included verbatim transcripts from in-person interviews and phone calls as well as from written responses to questionnaires sent to some of the study participants. Textual data were analyzed after de-identifying the data. Coding and data analysis were carried out using qualitative data analysis software, MAXQDA, Version XII [28]. Codes were used for each of the key themes and subtopics that emerged as the data was analyzed. Codes were revised and refined as new themes and topics emerged during the data analysis. A single researcher carried out the coding and analysis, ensuring no discrepancies in the use of codes. Inter-coding agreement of a part of the data and comparison of the use of codes was done to ensure reliability, and to resolve any discrepancies in coding. An independent translation company translated and transcribed the Spanish interviews and recordings from Argentina and Mexico into English.

The primary purpose of the qualitative data analysis was to identify themes or patterns in the responses to the research questions that would be included in a review of STs. A theme represented a pattern in the responses to each question and emerged from the coding of the data. All emergent themes and sub-themes were considered regardless of the frequency in the data set. A thematic analysis was conducted by developing detailed descriptions of the important issues around the use of STs for confirming medicines quality and detecting SFs. To compare common themes and patterns across interviews, data was grouped according to the country in which the interview took place.

## Results

Of the 53 interviews carried out for this study, 32 were in-person (Argentina, India, Mexico, Nigeria, Zimbabwe), 16 were phone interviews (China, Egypt, India, Jordan, Philippines, USA), and 5 were written questionnaires (China, USA). Results of the analysis showed a wide variation in understanding and usage of STs in different sectors. Overarching themes included a need for current information and data on the capabilities of available STs, the importance of transferable spectral libraries, lower acquisition and maintenance costs, simpler training requirements, access to in-country maintenance and technical support for technologies, as well as expectations and technical suggestions for the next generation of STs.

While most information related to the use of STs is summarized in Tables 2–4, additional information was collected throughout the course of the interviews. This information is included in Additional file 2 and Additional file 3. The literature review results were used to develop the background, formulate the interview guides, identify themes related to the use of ST’s and determine the types of personnel to be interviewed. Therefore, the results section focuses on the interviewees’ thoughts and perceptions related to the use of ST and poor quality medicines in their respective countries.

**Table 2:** Current Surveillance Practices and Quality Control Trends

Region	Government Regulator	Quality Control (QC) Trends	Surveillance Practices
Africa	Nigeria		
	▪ Nigeria – NAFDAC	▪ Preliminary screening, spot checks, and audits are conducted after products are imported, registered, and placed in market circulation. ▪ SF medicines detected: antimalarials and paracetamol are often found to be falsified.	▪ NAFDAC quality control laboratories support surveillance functions; local pharmaceutical companies, distributors and pharmacies ensure medicines are procured from trusted sources and perform occasional supplier spot checks.
	Zimbabwe		
	▪ Zimbabwe – MCAZ	▪ Manufacturers perform visual check of packaging only after products are released to the market. ▪ Interviewees noted difficulties sharing WHO alerts about poor quality medicines with other Southern African Development Community countries.	▪ MCAZ must purchase samples for analysis. ▪ MCAZ receives alerts, holds suspect product, and contacts the manufacturer, suppliers, and wholesalers; alerts pharmacies as to specific batch numbers.
Americas	Argentina		
	▪ Argentina – ANMAT	▪ Distributors and pharmacies rely on the National Traceability System for assuring product quality and deterring SF medical products. ▪ Some manufacturers request that quality control laboratories test their starting materials and finished products. ▪ A manufacturer performs packaging inspections via microscope and infrared to determine the authenticity of suspect products.	▪ ANMAT inspectors collect drug product samples at private and public hospital pharmacies, distributors, wholesalers, drug stores, and customs; and conduct inspections at manufacturers. ▪ The Traceability Resolution mandates, a national system for integrated, unique identifiers of specific medicines. ▪ Raw materials and finished products are bar coded, but PMS is not done on products after they are sent to a distributor^a^. ▪ A manufacturer uses a two-dimensional data matrix system and product serial numbers to track batches to the wholesale supplier and pharmacy.
	Mexico		
	▪ Mexico – COFEPRIS	▪ A lack of standard quality control equipment and a high volume of analysis are major challenges for laboratories. ▪ COFEPRIS’s OMCL analyzes manufacturer-produced raw materials and finished products classified as controlled medicines; and authorizes destructionof poor quality products.	▪ The monitoring division of the COFEPRIS sends inspectors to manufacturers, distributors, and point-of-sale drug stores to monitor their compliance with quality regulations. ▪ An operations division carries out enforcement actions related to non-conforming medicine samples.
	United States of America		
	▪ USA – FDA	▪ Multinational drug manufacturers use detection technologies (e.g., handheld spectrometers) at their plants to identify counterfeit raw materials. ▪ Ion Mobility Spectrometers are used for screening dietary supplements; Counterfeit Detection Device Version 3 for suspect products.	▪ FDA performs surveillance and targeted testing of samples for product and package evaluation at importation locations and FDA laboratories. ▪ The Drug Enforcement Agency and Customs and Border Protection carry out screening activities on medicines and health products.
Eastern mediterranean	Egypt		
	▪ Egypt – NODCAR	▪ Active pharmaceutical ingredients are imported and are verified prior to production; excipients are not verified but rely on internal vendor qualification procedures^b^. ▪ Pharmacies purchase products from trusted registered distributors and wholesalers.	▪ PMS focuses on medicine storage and stability. ▪ NODCAR conducts visual inspection of packaging in random market sampling; suspect products are sent for laboratory testing. ▪ The Directorate for Quality inspects imported products; samples are sent to quality control laboratories for testing.
	Jordan		
	▪ Jordan – JFDA	▪ Lack of human, instrumental, and financial resources are major challenges for the JFDA laboratory. ▪ A manufacturer uses bar codes on packaging as the only PMS and security checks of its products on the market.	▪ JFDA leads PMS activities and performs sampling and analysis at a single quality control laboratory. ▪ Medicines deemed suspicious by customs are quarantined until JFDA laboratory test results confirm product quality.
Southeast Asia	India		
	▪ State FDAs	▪ State FDAs noted the following challenges: ▪ Acquiring reference standards and analyzing samples quickly. ▪ Acquiring, maintaining, and funding recurring costs associated with laboratory equipment. ▪ A manufacturer noted: ▪ Performing periodic tests on random samples from its warehouse. ▪ Monitoring temperatures for proper transportation and storage conditions throughout the supply chain. ▪ Using tamper-resistant seals, barcodes, and pharmaceutical codes on its products. ▪ Pharmacies noted: ▪ Using no STs to monitor shelved products. ▪ Using online inventory, bar code scanning, and checking tablet codes for product quality. ▪ Obtaining products from manufacturer to avoid SF products. ▪ Relying on local Food and Drug Control laboratory for testing products that receive complaints.	▪ State FDAs have primary responsibility for screening activities. ▪ Inspectors sample medicines from sites (e.g., retailers, wholesalers, hospitals, clinics) at various geographic locations. ▪ Each quarter, certain classes of medicines are earmarked for screening. Samples are tested for identity, potency, and disintegration; results trigger enforcement action. ▪ Some state FDAs use a public portal as part of its e-Governance platforms for pharmacovigilance, SF notices, and recalls. ▪ A pharmaceutical company with well-equipped laboratories performs PMS on its products prior to release to market.
Western Pacific	Philippines		
	▪ Philippines FDA	▪ Philippines FDA laboratory in Alabang typically performs quality control testing. ▪ Manufacturers in the Philippines do not perform PMS on their products. ▪ Equipment for testing products within the supply chain is lacking; however, visual inspection of packaging is performed^c^.	▪ Philippines FDA conducts surveillance and screening activities. ▪ Field inspectors visit and collect samples from drug stores, pharmacies, and distributors. ▪ FDA laboratory conducts compliance testing of medicines received by the Department of Health central warehouse from suppliers.
	China		
	▪ The National Institutes for Food and Drug Control (NIFDC)	▪ Sample products are submitted to the China Food and Drug Administration to ensure adherence to current good manufacturing practices and guidelines from the International Conference on Harmonization of Technical Requirements for Registration of Pharmaceuticals for Human Use, according to a manufacturer. ▪ Pharmacies noted that pharmaceutical products are: ▪ Obtained from qualified suppliers ▪ Visually inspected and batch numbers checked for product quality ▪ Stored under recommended conditions; ▪ Consumers can check products for the manufacturer name, batch number, and China Food and Drug Administration certification number.	▪ NIFDC performs annual quality assurance including random checks of medicines and other medical products. ▪ Local NIFDC performs city-level PMS and checks for SF medicines by sampling products from rural hospitals and drug stores. ▪ Essential medicines obtained from the market are tested in mobile vans with instruments that can be linked to a laptop for data transfer. ▪ Manufacturers lack the resources or capability to carry out routine PMS^d^. Most SF products found at the pharmacy are damaged products, which are sent back to the suppliers and if this happens “too often” the supplier is changed.

^a^According to a manufacturer

^b^According to a manufacturer interviewed

^c^According to a DOH provincial hospital pharmacy

^d^According to a manufacturer interviewee

**Table 3:** Screening Technologies – Current Use, Benefits, and Limitations

Region	Screening Technologies Used	Benefits	Limitations
Africa	Nigeria		
	▪ Thermo Fisher Scientific’s Handheld Raman, TruScan ▪ Global Pharma Healt Fund’s Minilab	▪ Pharmacies support the use of STs to detect SFs. ▪ TruScan use was seen as reducing SF prevalence. ▪ Minilab primarily used to check antimalarial medicine quality.	▪ TruScan’s associated cost; reference standards requirements; time for library development; inability to work through packaging; software ease of use; training requirements; after-sales service; maintenance; and repairs. ▪ Minilab cannot perform dissolution testing; results were not sufficiently quantitative, limiting its use in litigation. ▪ Pharmacies, manufacturers, and distributors were not using ST; most felt that NAFDAC was responsible for assuring the quality of medicines within the local marketplace ▪ Manufacturers indicated that cost and the lack of available information on STs limited their uptake.
	Zimbabwe		
	▪ Thermo Fisher Scientific’s Handheld Raman, TruScan	▪ TruScan spectrometers employed to detect SF at some ports of entry. ▪ MCAZ noted that Minilabs would be particularly useful at the ports of entry. ▪ Ministry of Health would like to place TruScan units at all ports of entry and across the supply chain if funding were available.	▪ Although regulators, manufacturers, and pharmacies expressed interest in using portable STs, none reported widespread use. ▪ Most cited lack of ST affordability as a limiting factor; some, insufficient information about comparing available technologies.
Americas	Argentina		
	▪ Raman spectrometers used by a manufacturer for its quality control of raw material and finished product active pharmaceutical ingredient identification ▪ Thermo Fisher’s Handheld near infrared spectrometer, Phazir used by a manufacturer for identifying starting materials	▪ Raman spectrometers save reagents and identify material outside of the quality control laboratory; this speeds operations and prevents contamination because there is no need to open multiple drums. ▪ NIR is fast and simplifies product identification.	▪ Lack of information on availability and cost of non-Raman technologies; difficulties procuring and importing new technologies^a^. ▪ A manufacturer did not use any STs, but relied on testing by an anti-counterfeit laboratory focused on product security in the Latin American region. ▪ Pharmacies do not use STs; they rely solely on the National Traceability System to ensure medicine quality^b^
	Mexico		
	▪ Handheld near infrared spectrometer was used by a manufacturer. ▪ STs are not used by COFEPRIS; pharmaceutical analyses are currently done in laboratory by compendial methods. ▪ STs were not used by distributors or pharmacies.	▪ Handheld near infrared spectrometer is used for API identification during production; this manufacturer was evaluating the benefits of near infrared compared with Raman. ▪ Raman has the broadest scope of use based on the chemical characteristics related to the components used by the manufacturer. ▪ ST awareness is expected to grow under new legislation that manufacturers need to identify raw materials, active ingredients, and excipients in containers unless the vendor has been pre-qualified.	▪ COFEPRIS does not recognize ST technologies for confirming medicines quality because the Mexican Pharmacopoeia does not recognize these methodologies for testing or have a standardized way to deploy or interpret the results.
	United States of America		
	▪ Raman, alternative light source, and Ion Mobility Spectrometers .	▪ ST technologies are validated prior to field use. ▪ Alternate light source and Ion mobility spectrometers have successfully detected SFs entering the U.S. ▪ Raman spectroscopic tests are well characterized, with accumulated knowledge on how to use them in quality testing; instrument vendors provide training; maintenance is low, simple, and affordable. ▪ Raman and near infrared distinguish among organic substances, inactive ingredients, and most active ingredients in pharmaceuticals and final products.	▪ Maintaining and updating spectral libraries are the biggest challenges the FDA faces. ▪ The cost of commercial devices is high compared to in-house developed alternate light source technology. ▪ Using Raman to test certain products with fluorescence and low-dose concentrations is problematic without further offline processing of results. ▪ Raman cannot reliably authenticate all of its medicines, a manufacturer commented; the results depend on the complexity of the chemical composition. ▪ Independent pharmacies in one state do not use any STs, citing expensive ST instrumentation and a perception that the quality of commercially available products has already been ensured.
Eastern Mediterranean	Egypt		
	▪ Handheld STs are not used by regulators or manufacturers. ▪ Pharmacies do not use STs, but some use two-dimensional barcoding.	▪ STs for raw materials that are imported into Egypt, some of which have been substandard in the past, would help the manufacture of products for domestic consumption^c^.	▪ Reasons for not using STs included: ▪ Lack of knowledge about the benefits of STs ▪ ST training costs ▪ ST results are insufficient grounds for taking action against a poor quality product; full compendial laboratory analysis is necessary. ▪ SF medicines are perceived as too low to justify purchase and use such of ST, according to a regulator; STs would probably not improve PMS activities or the ability to detect poor quality medicines. ▪ STs are affordable for multinational pharmaceutical companies but not for smaller manufacturers, according to a manufacturer.
	Jordan		
	▪ STs are not used by the JFDA. ▪ Handheld Raman and near infrared are used for identification of raw materials by Jordanian manufacturers. ▪ STs are not used at the pharmacy; however, barcoded pricing is obligatory and can trace products from the pharmacy shelf back to the manufacturer.	▪ JFDA quality control laboratory noted that handheld ST devices could reduce overall workload of analysts, but preferred that the purchase of traditional lab equipment instead of “costly” STs. ▪ Near infrared spectrometers were cheaper and safer for users than other currently available STs; and the vendor provided training.	▪ Challenges JFDA faced when it did use ST (TruScan) included: limited spectral library; short battery life and lack of a backup battery; large size; lack of touchscreen function; high cost; and lack of in-country customer service. ▪ JFDA cannot use ST results as evidence of SFs in reports.
Southeast Asia	India		
	▪ State FDA used mobile testing vans equipped with Thermo Fisher Scientific’s MicroPhazir RX 4.0 handheld near infrared; SciAps Inspector 300 Raman spectrometer; and Elvatech Pro X-Ray Fluorescence spectrometer. ▪ vHandheld Raman and near infrared spectrometers were used by Customs. ▪ Handheld near infrared spectrometers were used by manufacturers; internal spectral library was shared with other manufacturing facilities.	▪ Regulators and manufacturers noted that: NIR spectrometers are easy to use, non-destructive of the dosage form, and work through packaging; Raman spectrometers are lightweight and fast; after spectral libraries have been developed, they can be used to analyze samples quickly. ▪ State FDA is considering using automated sterility, microbial enumeration, and microbial identification systems to reduce laboratory time testing products. ▪ Manufacturers noted that spectral libraries are product-specific, and some raw material spectra can be shared easily.	▪ Regulators and manufacturers noted that: ▪ Key limitations of STs relate to spectral library development, maintenance, expansion, and sharing. ▪ Near infrared spectrometers cannot be used for fixed-dose combination products; on certain coatings; for low-dose products; or for differentiating structurally similar compounds (e.g., azithromycin, erythromycin). ▪ The Raman spectrometers cannot be used for detecting fluorescent compounds or water-based liquid dosage forms; and they do not have peak labeling capabilities. ▪ Manufacturers noted that results obtained with STs are only preliminary. Additional challenges include the complexities of validating and calibrating the technologies and training requirements for staff. ▪ STs were not used by pharmacies that participated in interviews; limiting factors include the cost of ST technology, training, and the developing a quality control system.
Western Pacific	Philippines		
	▪ Global Pharma Health Fund’s Minilab	▪ Philippines FDA sentinel sites use Minilab for initial screening of medicines from retail outlets. ▪ There are plans to provide Raman spectrometers or handheld X-ray fluorescence spectroscopy to regional field offices for product screening. This would enable Philippines FDA laboratories to maximize their resources because only ▪ Confirmatory testing of suspect pharmaceutical samples would be necessary.	▪ Challenges of using Minilab include: ▪ Tracking the supplies and importing certain reagents needed to run tests^d^. ▪ Training FDA and Department of Health staff to use Minilab, which is compounded by frequent staff turnover.
	China		
	▪ Thermo Fisher Scientific’s Handheld Raman TruScan is used by manufacturers. ▪ Handheld near infrared and Raman spectrometers are carried by NIFDC mobile vans; they also carry smaller versions of lab-based high performance liquid chromatography.	▪ Mobile vans and handheld spectrometer technologies do not require chemical reagents. ▪ High performance liquid chromatography testing uses an abbreviated, quick result protocol; suspicious samples are sent to a quality control laboratory for full compendial testing. ▪ APIs and finished pharmaceutical product manufacturers send numerous samples to provincial regulators for spectral library development; and an annual report with this screening data is prepared for the Chinese Food and Drug Administration.	▪ Development of spectral libraries is a major challenge associated with the use of near infrared and Raman. Spectral libraries are not shared among provinces, which makes communicating findings between provinces a challenge. ▪ Training is an issue because vendors conduct training at the time of purchase. ▪ STs are not used by manufacturers or pharmacies to screen finished products in the market.

^a^Manufacturers noted

^b^Pharmacies noted

^c^According to a manufacturer

^d^According to a regulator interviewed

**Table 4:** Screening Technologies – Ideal Qualities

Region	Ideal Qualities
Africa	Nigeria
	STs should be: ▪ battery powered ▪ easy to use ▪ fast ▪ portable ▪ provide high sensitivity and specificity results ▪ quantitative ▪ reliable ▪ run microbiological and impurity profiling tests
	Zimbabwe
	STs should be: ▪ accessible ▪ centralized and automatic data back-up ▪ easy to calibrate ▪ easy to use ▪ employ non-destructive sampling ▪ able to follow U.S., British, or International Pharmacopeia test methods ▪ inexpensive ▪ offer local access to technical support ▪ provide instant reporting ▪ quick setup time
Americas	Argentina
	STs should: ▪ determine levels of impurities ▪ employ non-destructive sampling ▪ perform instant microbiological counts
	Mexico
	STs should be: ▪ compact ▪ cost-effective ▪ easily interpretable results ▪ easy to calibrate ▪ easy to use ▪ employ non-destructive sampling ▪ equipped with a camera and/or barcode reader ▪ have the capacity to test all products without having to develop a labor-intensive spectral library ▪ inexpensive ▪ portable ▪ provide high sensitivity and specificity results ▪ capable of rapid analysis ▪ generating results comparable to standard compendial analyses (i.e., limits of detection, quantification) with the ability to extract the data
	United States of America
	Surveillance and screening technologies should be: ▪ able to analyze both product and packaging ▪ easy to maintain ▪ easy to use ▪ capable of big data analysis and linkage ▪ inexpensive ▪ rugged
Eastern Mediterranean	Egypt
	STs should be capable of: ▪ providing qualitative results of active pharmaceutical ingredients prior to production ▪ testing most excipients
	Jordan
	STs should: ▪ provide accurate results ▪ have the capacity to check the quality of finished products ▪ be handheld ▪ have a touchscreen ▪ include an extensive and validated spectral library ▪ include in-country customer service ▪ be inexpensive ▪ have long battery life ▪ provide quantitative results ▪ support all pharmaceutical products (i.e., not just one manufacturer’s products)
Southeast Asia	India
	Mobile vans should include: ▪ ability to conduct rapid on-the-spot analysis of medicines ▪ disintegration tests ▪ high sensitivity and specificity technologies ▪ high performance liquid chromatography ▪ staffing by an analyst, inspector, and a laboratory assistant
Western Pacific	Philippines
	STs should: ▪ provide accurate results ▪ include customer service ▪ provide data that are transferable to computers without requiring other systems/software ▪ be easy to maintain ▪ provide faster quality control testing of raw materials and finished product throughout its shelf life ▪ be inexpensive ▪ be reasonable in size and weight ▪ provide results comparable to those of benchtop instruments ▪ be sensitive ▪ be specific ▪ be user-friendly
	China
	STs should: ▪ include barcodes ▪ be compact (i.e., suitcase size) ▪ be easy to maintain ▪ follow U.S. Pharmacopeia monograph specifications ▪ be handheld ▪ include in-country customer service ▪ be inexpensive ▪ have long life span ▪ provide rapid screening results ▪ be robust ▪ use less organic solvents

Table 2 summarizes the information provided by regulators, manufacturers, pharmacies and distributors on their current screening and quality control practices.

All of the regulators and manufacturers interviewed have quality control laboratories to assure product quality either prior to release, in the case of manufacturers, or post market, in the case of regulators. Understandably, none of the distributors or pharmacies have quality control laboratories. Qualification or vendors and documentation checks are therefore used by these organizations to assure product quality of incoming samples. While most regulators conduct some form of post market surveillance, in countries where screening technologies are not being used all of these samples must be tested at the quality control laboratory.

Table 3 provides information on current use practices related to screening technologies as well as the benefits and limitations interviewees are facing in the deployment of these technologies.

Handheld Raman and near infrared spectrometers are commonly used by manufacturers primarily for raw material screening, while some regulators are using Minilab™ in addition to handheld Raman spectrometers as part of their post market surveillance programs. Those regulators not currently using STs cited cost as one of the major prohibitive factors while others highlighted that screening results cannot be used for regulatory action.

Table 4 summarizes the feedback provided by interviewees on the ideal qualities of a screening technology. Table 5 provides acronyms used throughout this article.

**Table 5:** Acronyms

MRA	Medicine Regulatory Authority
OMCL	Official Medicines Control Laboratory
PMS	Post-Marketing Surveillance
SF	Substandard and falsified
ST	Screening technology
WHO	World Health Organization

These results indicated a trend toward the following characteristics deemed ideal for a screening technology; compact size and easy to use, calibrate and maintain, fast and reliable with greater sensitivity and specificity and an ability to provide quantitative information, transferable data libraries, simple training requirements and finally, in-country technical support.

## Discussion

The data obtained from the 10 countries selected in this study have shown wide variation in the awareness, understanding, and usage of medicine quality screening technologies in different sectors around the world.

### Regulatory authorities

While regulators in China, India, Nigeria, the Philippines, and the US have been using one or more STs for several years, regulators in Argentina, Egypt, Mexico, and Zimbabwe are not using these devices. Jordan was using one ST for years but stopped. Interviewees provided a variety of reasons for using or not using these technologies. While Nigeria and the USA import many of their medicines and deploy screening technologies at their borders, other countries like India and China focus their efforts more on identifying poor quality medicines already in circulation in their local formal and informal markets. According to a regulator in China, “we collect samples nationwide from the market and they [inspectors] bring the collection back to the lab and test them.” All of the countries surveyed have informal markets where SF versions of essential, common, fast-selling, as well as expensive, low-volume medicines are found at cheaper prices. If inspectors had access to affordable, easy-to-use, handheld STs with analytical techniques recognized by the local MRAs, rapid, evidence-based decisions could be made about suspect products in these settings. The deployment of mobile labs in China and India is an excellent success story and has provided valuable expertise and flexibility to regulators keen on facilitating quick quality analyses and taking appropriate regulatory actions. In the USA, screening using handheld detection technologies at import locations and international mail facilities, immediate SF alerts, and sharing of information between domestic and international regulators has been successful in rapidly detecting poor quality medicines. These two examples provide a possible framework for large and small countries alike that are eager to incorporate STs into their quality control and assurance systems.

Regulatory authorities in nine out of 10 countries believe that screening technologies can and do enable more efficient and risk-based PMS by increasing the volume of samples that can be rapidly screened in field settings and reducing the volume of samples that must be transported to and tested at quality control laboratories; thereby increasing coverage and decreasing the overall cost of surveillance. However, the effective deployment of STs is predicated on an understanding of their capabilities. As such, most regulators also expressed an interest in having information about the capabilities of existing STs to understand what is out there and inform their future procurement decisions.

### Manufacturers

Contrary, but complementary to regulators, manufacturers deploying STs were using them almost exclusively to confirm the identity of raw materials. It is impossible for manufacturers that produce a large variety of products to test every container of active ingredient and excipients that they receive using compendial methods. Preferred technologies such as handheld Raman and near infrared spectrometers therefore provide quick and reliable qualitative identification information about these materials. A U.S. manufacturer indicated that these “peer-reviewed technologies are acknowledged as accurate, effective, and suitable as predicates for expert testimony in court.” Deployment of STs is also in response to most regulators requiring manufacturers to inspect their raw materials used in production. As pharmaceutical manufacturing regulations in developing countries begin and continue to incorporate Quality by Design principles, which refer to manufacturers understanding and subsequently designing a manufacturing process that consistently delivers the desired product quality (FDA 2006 – Guidance for Industry, Q8 Pharmaceutical Development), we would imagine that manufacturers in these settings might start to incorporate STs into their systems even more [29].

The absence of routine PMS by manufacturers in most of the countries surveyed means that only products that receive consumer complaints get checked for quality. Although a Philippine manufacturer is interested in deploying screening technologies “to check incoming raw materials and to use it in the finished product line”, several manufacturers in lower and lower-middle income countries included in this study felt that it is the responsibility of the MRA to monitor post market medicine quality. The authors of this article challenge this perception because the reality of MRAs in lower income countries is one of being overburdened and underfunded. It would therefore behoove these manufacturers, particularly those from China and India that are exporting great quantities to these lower income countries, to consider implementing their own PMS programs. Firstly, by demonstrating a commitment to PMS they would be projecting transparency and a reputation for accountability to their customers, which may indirectly drive sales through an increase of ‘brand trust’. Concomitantly, data acquired through PMS could be shared with local MRAs to foster collaboration, detect problems with vendors, and identify systemic issues within local supply chains.

### Pharmacies and distributors

Pharmacies and distributors in all 10 countries stressed that the STs they are aware of are cost prohibitive and some distributors mentioned the need for extra space to carry out screening activities as a drawback. Therefore, they rely heavily on track and trace technologies and complement these approaches with good procurement practices and documentation checks to maintain customer confidence. As one pharmacist in Argentina said, “Our main tool is the evaluation of suppliers and clients…whose products we will be selling.” Track and trace systems can verify the authenticity of a product but cannot determine whether possible improper storage and transport have compromised the potency or even the identity of the active ingredient. We believe STs can elegantly complement the track and trace technologies currently in use; particularly in countries where the supply chain is fragmented, and high heat and humidity contribute to medicine degradation. It would be interesting to understand further how pharmacies and distributors define and quantify ‘cost prohibitive’, whether their procurement practices and existing checks incorporate elements of risk management or cost-benefit analysis, and how the role of STs could mitigate risk and reduce cost of their operations in the long term.

Users need to be able to determine the benefits and identify the limitations of using specific STs. Interviewees identified constraints to using STs, which included cost, development of spectral libraries, limited human resources and trained personnel, and availability of technical support and customer service.

### Screening technologies—Benefits, limitations, and the ideal instrument

Virtually all of the regulators, manufacturers, pharmacies, and distributors indicated a lack of trustworthy, accessible information on screening technologies to inform them on what technologies would be beneficial for their needs. In fact, through their responses, a number of participants demonstrated a lack of understanding about the capabilities of existing STs. This reinforces the need to have information on types of STs as well as clear explanations of how they function. The information should be at a level that is understandable not only by highly technical quality control lab staff but also regulators, MOH officials who make high-level budgetary decisions, customs agents, distributors, pharmacists and even patients. Therefore, a key recommendation from this study is the need for a standardized, trustworthy, scientifically sound, and comprehensive technical review of the capabilities of current STs that is accessible to any organization interested in using these STs to reduce the prevalence of poor quality medicines globally, regionally, and locally. These reviews should also be objective, in that they are performed by an independent organization with technical expertise on the techniques that underpin the technologies of interest. This would provide valuable information to current and potential users to identify technologies that are most suitable for their particular requirements.

Several interviewees indicated that objective reviews of STs would be valuable to inform existing and potential technology users and guide the direction of future quality control testing activities. A regulator from China said reviews would be useful to know what is being used outside of the country while a Zimbabwean regulator expressed that this information would allow them to assess and compare technologies and select those most appropriate for their needs. However, comparative or ranked reviews of technologies should be avoided when evaluating technologies that employ different techniques. In the same way that Fourier Transform Infrared spectroscopy should not be compared to High Performance Liquid Chromatography, a handheld Raman instrument should not be compared to an alternate light source instrument. The technologies provide complementary information, and while one may be more suitable in a particular context or setting, it would be inaccurate and misleading to categorically state that one is ‘better’ than another.

The context for implementing STs varies across user groups and, as such, may require additional tailored work, context-specific research, and targeted evaluations. For example, a manufacturer seeking to identify a few raw materials in the relatively controlled setting of a receiving warehouse may require a different technology than an inspector sampling and screening hundreds of products in rural pharmacies where temperatures are high and power sources are not available. In another setting, patients in urban areas would benefit from an internet-connected, smart phone-compatible device that provides a simple yes-no result. Conversely, a global procurement agency conducting quality control analyses after receiving a shipment might require multiple, more complex technologies that can quantify the active ingredient of interest, detect impurities, and measure dissolution. In fact, global health and donor organizations are integral in assisting lower income and lower-middle income countries to obtain, pilot, maintain, and train staff for these STs. Significant international aid is given to procure medicines and it is often a requirement and always in the donor’s best interest to ensure the quality of these donated medicines is maintained throughout the supply chain as exemplified by the Global Fund’s Quality Assurance Policy, which requires that the “source and quality of the raw materials entering into the finished product meet accepted quality standards” and that “quality control measures are in place and adequate” for all pharmaceutical products procured with Global Fund money [30]. A more specific example of the value of post-marketing surveillance can be seen in Liberia, where with support from the U.S. Agency for International Development-funded, U.S. Pharmacopeia implemented Promoting the Quality of Medicines program, the Liberian Medicines and Health Products Regulatory Authority used Minilab™ and confirmatory testing to show that half of antimalarial medicines sampled in a 2010 and 2011 study were of poor quality [31]. Therefore, the international donor community should be further engaged in funding and supporting countries to select STs and implement their use.

Although interviewees stressed a lack of objective information about the available STs and the fact that “there is no one perfect technology”, most regulators were able to identify the features of an ideal technology for their specific settings as highlighted in Table 4. In some instances, Minilab™ has been replaced by Raman and near infrared spectrometers because these do not require chemical reagents for testing. Users of one technology were reluctant to acquire a different technology after investing significant time in its calibration and maintenance and the development of customized spectral libraries. Low-maintenance instruments are therefore preferred because service providers and instrument vendors are often not located in the country and it is costly to schedule travel for servicing and repair of inoperable instruments. In the Philippines, where Minilab™ is being used for surveillance and screening, procuring and importing reagents for the kits requires special permits from law enforcement. Therefore, many of the products in informal markets in the remote areas of the Philippines do not undergo adequate and sufficient screening. This is a challenge faced in many remote areas of the developing world so the ability of a technology to operate effectively without the need for large amounts of consumables and reagents is critical in these settings. Expanding upon this, many interviewees stressed the importance of a screening technology that is easy to transport (i.e., can be carried by one person, as small as a briefcase), rugged, and simple to train staff on and use. According to one of the pharmacists interviewed in Zimbabwe “[a device] should be simple and practical such that it can be integrated into our current receiving systems. For example, the dispatch clerk or a warehouse manager can actually use it, where it won’t need another specialized individual to come on board. This technology should help us understand the extent of the problem.”

A technology that provides quantitative information about the active ingredient was also mentioned several times as a need and instrument manufacturers should heed this recommendation. Minilab™ provides semi-quantitative information but is very operator dependent, meaning the accuracy of the results depend on the user’s skill level and familiarity with thin layer chromatography. Contrary to this, most handheld spectrometers are generally operator independent when performing identification tests, but at the very least, require complex offline chemometric analysis to quantify the active ingredient. The example included in the results section can be used by instrument manufacturers to continue to refine their technologies and to provide the support that goes along with them.

Regulators, manufacturers, distributors, and pharmacies all highlighted the limited information available on the financial resource requirements of procuring and deploying STs. These requirements encompass not only the upfront price of a technology but also cost per test, cost of consumables and accessories, cost of calibration and maintenance, and the human resource needs. Regulators especially, but also manufacturers, distributors, and pharmacies, should budget for all these items in the use of STs for PMS and/or quality assurance and quality control activities. Very few of the stakeholders interviewed had financial resources to purchase and ‘pilot’ instruments. Because of limited financial resources, as well as the time spent in the initial stages of procuring and deploying any given technology, access to practical budgetary information in addition to performance capabilities is invaluable in assisting users to identify how effective and sustainable a given technology is prior to procurement.

One idea that emerged from this study is the potential for ST equipment manufacturers to establish a tiered pricing mechanism similar to what some pharmaceutical manufacturers do with medicines. For example, the cost for a low-income country to purchase a specific handheld spectrometer would be A, while in a high-income country it would be C, and in middle-income countries it would be B, somewhere between A and C. In many countries, procuring equipment from outside the country and obtaining permission from the government to import these instruments requires tremendous amounts of paperwork and time. A manufacturer in Argentina stressed that “you have to meet hundreds of legal requirements and criteria just to get them [customs] to consider allowing equipment to enter the country.” Attempts to obtain the necessary permissions are often met with failure to acquire the technology needed. In Egypt, Jordan, and Mexico, regulators and manufacturers wanted to see detailed guidelines for the use of specific STs and acceptance of these technologies by the MRAs as a permissible form of testing, from which results could be used to prompt recalls and enforcement action. Currently, the absence of such recognition by either governments or pharmacopeias is one of the limiting factors for regulators to deploy these technologies at ports of entry. More specifically, the analytical techniques used by many STs are generally not the techniques accepted by the recognized local or international standard. Although certain MRAs have established standards that enable enforcement action on the basis of screening technology results, further legal and regulatory recognition would facilitate the incorporation of technologies such as handheld Raman or near infrared spectrometers into their existing PMS programs. Therefore, users must take into consideration not only costs and import restrictions, but also regulatory recognition of technologies as part of the process of medicine quality testing and subsequent enforcement action against poor quality medicines.

These sentiments reflect divergent thoughts of interviewees about the role of STs. With the advent and increasing uptake of continuous manufacturing, there has been fervent discussion about process analytical technology—which often includes STs such as handheld spectrometers—and its role in redefining, or controversially, replacing traditional quality control testing [32]. Certainly, as this new paradigm continues to evolve, the ubiquitous validation and recognition of screening technology test results to prompt legal action may become a reality. However, for the time being, quality control testing and the laboratory-based techniques it encompasses will remain the primary method for MRAs to obtain the data needed for enforcement action. Presently, screening technologies are just that—screening technologies. There is no ‘silver bullet’ technology so multiple STs are often, if not always, needed to paint a complete initial picture of the quality of a medicine. They should be used to conserve the resources of, reduce the burden placed on, and drive the sustainability of OMCLs and help manufacturers, distributors, and pharmacies confirm the quality of medicines they are producing, procuring, and selling. They are the integral second part of Pribluda et al.’s Three-Level Approach [33, 34]. An excellent example of this is in Nigeria, where the use of multiple handheld STs has reduced the prevalence of SFs in circulation. Nigeria’s National Agency for Food and Drug Administration and Control has incorporated the use of handheld STs in their government quality surveillance activities at the borders and at sentinel sites throughout the country and the use of results for enforcement after further confirmatory testing.

Although only 10 countries were included in this study, they represented five of the six WHO geographic regions and all World Bank economic classifications. This was done deliberately to ensure that the perspectives, successes, and challenges from a variety of settings were identified. This will also allow countries that were not included to glean information about settings similar to theirs and to identify opportunities from settings dissimilar to theirs. In-depth interviews of over 50 participants across three categories of organizations enabled the qualitative researcher to reach saturation. Therefore, we are able to provide a deeper and more robust understanding of SF perceptions and ST use rather than focusing solely on one type of organization, given that regulators have different needs than manufacturers, pharmacies, and distributors. However, it would be useful to re-visit some of the countries surveyed to see if after acquiring new technologies their experiences mirror the experiences of the countries that were already using such technologies. For instance, Zimbabwe was in the process of procuring handheld Raman spectrometers to use at their ports of entry while Nigeria has used these technologies at their ports of entry for almost a decade. Will Zimbabwe’s MRA experience the same issues that were faced by Nigeria or will Nigeria’s challenges and successes inform and enhance Zimbabwe’s activities? In this vein, this article can drive collaboration between organizations to share best practices and experiences.

### Limitations

While this article has generated novel data and information on the topic of ST use, there are limitations to the paper. Firstly, Europe—the sixth WHO region, was not included in this study, which we consider a limitation. The focus for country selection initially was on low, middle, and upper-middle income countries with available SF data; the included Organization for Economic Cooperation and Development and high-income countries (USA, Mexico, and Argentina) were added based on U.S. Pharmacopeia contacts and feasibility to facilitate interviews. Secondly, difficulties in contacting interviewees and scheduling time for visits and phone interviews meant that the data from a few countries was not as expansive as data from other countries. Thirdly, discussing SFs in all countries can cover sensitive topics in the public and private sectors, irrespective of whether one is a regulator, manufacturer, pharmacy, or distributor. Although interviewers took precautions to avoid the use of leading questions, the nature of the topics of discussion may have resulted in the introduction of some bias that influenced the response of participants from giving divulging sensitive information. We strongly believe we were able to engage participants who were willing to share their perceptions based on the confidentiality of the research methods. However, some countries were more reticent to openly discuss the issue of SFs.

## Conclusions

This is the first multi-country, multi-stakeholder landscape assessment that utilizes qualitative methodology to examine practices and perceptions related to the use of medicines quality screening technologies. Existing and potential technology users can benefit from this landscape assessment and use the information to better understand these available technologies and the real benefit they can provide. It is well documented that compendial testing of all sampled medicines by OMCLs in lower to middle-income countries is virtually impossible due to budgetary limitations and human resource constraints.

This article can be used to help regulators communicate and justify their needs to acquire and invest in new STs. It identified the need for objective, technical reviews of the capabilities of current STs that are accessible to organizations interested in using these technologies, while confirming that there is no ‘silver bullet’ technology. Interviewees also highlighted the need for information on the financial resource requirements of procuring and deploying technologies. To support this technical information, strategies to enable regulatory recognition of ST results, where appropriate, could engender the broader update of these instruments by regulators. ST manufacturers and other organizations supporting these activities can take into account these recommendations and some of the limitations of these technologies identified by the interviewees and take steps to address them to better support their stakeholders.

As more countries and users realize the value of these technologies and begin to invest in them, an ongoing dialogue between regulators, pharmaceutical manufacturers, ST device manufacturers, pharmacies and distributors, academia, and donors who may purchase these instruments will be paramount to ensuring their effectiveness and continued value.

Medicines quality screening technologies play a pivotal role along the medicines supply chain by assuring the quality of medicines that are manufactured, distributed, marketed, and prescribed. They are and will continue to be, part of the future in the fight to control the proliferation and public health impacts of SFs globally, particularly in developing countries.

## Additional files

Additional file 1: In-depth interview guide (approx. 60–90 min) for regulators. Interview guide template used for the interviews conducted with regulators (DOCX 21 kb)

Additional file 2: Screening technologies by country, government Regulator [R], Manufacturer [M], and Distributor/Pharmacy [DP]. Identifies the screening technologies currently being used by the organizations interviewed, grouped by region and country. (DOCX 42 kb)

Additional file 3: Additional interview information on use of screening technologies in countries surveyed. Highlights additional information gathered from interviews, which was not directly used in the results of discussion sections. (DOCX 27 kb)
