# Supplementary material for: Global landscape assessment of screening technologies for medicine quality assurance: stakeholder perceptions and practices from ten countries
Source: Global Health. 2018 Apr 25;14:43. doi: 10.1186/s12992-018-0360-y (PMC5922304; doi:10.1186/s12992-018-0360-y)
Supplement: Supplementary file 1 — In-depth interview guide (approx. 60–90 min) for regulators. Interview guide template used for the interviews conducted with regulators (DOCX 21 kb) [file 12992_2018_360_MOESM1_ESM.docx]

**Additional file 1. In-depth interview guide (approx. 60-90 minutes) for regulators.**

**I. Introduction**

Hello, my name is Ameena Nalim. This interview is being conducted on behalf of the United States Pharmacopeial Convention (USP). I would like to thank you for your participation in this interview.

It will help us better understand surveillance and screening technologies used in the regulation of medicines and other medical products in this country.

The interview will take about one hour and your participation is completely voluntary. If you don’t feel comfortable with a question or would like to end the interview, please let me know at any time. Whatever you say will be completely confidential. No names will be used in any publication. I would like to ask your permission to record our conversation so that I can accurately capture the points you share. Kindly read over the informed consent document.

May I record our conversation? Would you please sign informed consent?

Do you have any questions before we begin?

**II. Warm-up Questions**:

1. Tell me a little about your work and how it helps to promote the quality of medicines and health products?
2. Where does ____(this country) obtain its medicines and other medical products?

Probes: produced in country, imported, from multiple sources, informal market

1. Where do the people of ___(this country) obtain their medicines and other medical products?

Probes: public (centrally procured), private (private pharmacies) and informal (markets, etc.) sectors

1. How do you define and categorize poor quality medical and health products?

**III. Main Questions:**

**My next questions are about the quality of medicines:**

For this interview, I am using the World Health Organization definition for poor quality medicines, that is, substandard, spurious, falsely-labeled, falsified, and counterfeit medical products (SSFFC). For the remainder of the interview, I will be using the acronym, SSFFC medical products.

**Scope of the problem**:

1. What has been your company’s experience with SSFFC products in this country?
2. What are some reasons for the presence of poor quality (SSFFC) medicines?
3. About what percentage of medicines and health products are of poor quality versus of good quality?
4. What are some types of harm that have been caused by this problem in__(this country)?

Probes: Can you give specific examples? (fatalities, adverse reactions, treatment failure…)

1. How aware are people of the prevalence of SSFFC products in the market?

Probe: Is there awareness amongst health professionals, consumers, and physicians?

**Supply chain for SSFFC**:

1. Where do SSFFC medicines and health products come from?
2. Where can you find SSFFC medicines and health products in ___(this country)?
3. What types of SSFFC medicines and medical products are found in ___(this country)?

Probe: Can you name a few of them?

1. Which medicine or health product is most commonly found to be SSFFC?
2. Why do you think this is the most common SSFFC product in ___(this country)?

**Regulation/Registration/Licensing**:

1. What **challenges** do you face in the regulation of medicines and health products?

Probes: under-staffed, economic, bribery/corruption, lack of qualified/trained staff, government funding

1. Based on what we know about registration of products in your country, can you tell us about issues relating to **unregistered medicines** here?

**Quality control**:

1. Can you tell us a little about the QC lab?

Probes: Is there one or more, is there testing at more than one facility?

1. What can you tell us about the activities of the National Quality Control Laboratory?

Probes: what type of accreditation it has or does not have

1. What are the **challenges** faced by the National Quality Control Laboratory?

**Surveillance (testing) activities**:

1. Do you conduct surveillance activities on medicines and health products?
2. When do these surveillance activities take place?

Probe: registration, imports, post-market surveillance, adverse event reporting (pharmacovigilance), border smuggling/illegal importation, back log at MRA to register

1. Where does this screening and surveillance take place?

Probe: screening at different levels of the supply chain: at border-crossings, initial screening at manufacture, at wholesale distributer, at pharmacies, at patient point-of-contact, informal markets

1. If screening is taking place at more than one location, are the results being communicated between locations?
2. Are the testing practices the same between locations?
3. How often is surveillance and screening done?
4. On what types of products is this surveillance done?
5. What testing methodologies are employed when samples are taken for quality testing?

Probe: break down into laboratory-based techniques and field-testing screening techniques

1. What challenges do you have in using these testing methodologies?

**Tools and screening technologies in use:**

1. What tools and screening technologies are used for detecting SSFFC medicines and other health products?

Probe: handheld technologies, testing labs, Minilab™,

Use Table of technologies to check off items (skip to 32)

1. What are the reasons for not using surveillance and screening technologies?
2. Do you have any plans to use them?
3. Who decides what technologies to use for surveillance and screening?
4. How are the decisions made about which technologies to use?
5. Where are these technologies used for routine screening?

Probe: screening at different levels of the supply chain: at border-crossings, initial screening at manufacture, at wholesale distributor, at pharmacies, at patient point-of-contact, informal markets

1. What medicines and health products do you routinely screen using these technologies?

Probe: ARVs, anti-malarials, etc.

1. Why are these particular technologies being used?

Probe: costs, training, supplies, speed, no sample prep, no lab needs

Probe: Decided by visiting representatives from technology manufacturers (Fischer). From donor agencies/international development partners (donated technologies, ex. Minilabs™ from PQM)

1. What are the **limitations** for the use of these technologies for your surveillance and screening purposes? Probes:

Are they user-friendly?

Do they need to replace batteries too often in the field? Do they work well on battery power?

Do they work through the packaging? Do they work well?

Do you have good customer service? Do they have to be serviced often?

1. What can you say about the a**ffordability** of these technologies?
2. How are you **acquiring** these technologies?
3. What is the **skill level** of personnel using these technologies?

Probe: Is there any training involved for personnel to use these technologies?

Probe: Is the skill level sufficient, would you like to see it improved and how?

1. What are the **limitations** to testing in this country?

Probes: infrastructure constraints, workforce issues whether it be skill level or numbers of available personnel, political issues, government preferences, corruption

1. What testing practices have been most effective?
2. How do these technologies help to maintain quality standards in the market?

Probe: remove poor quality (SSFFC) medicines and medical products, put new policies in effect, penalties enforced

1. **How successful** are these technologies in helping to combat poor quality (SSFFC) material?

Probes: detects few, some, many, all, pre-sale batches of poor quality (SSFFC) medical products

1. Which surveillance and screening technologies **have not been successful** and why?
2. Overall, **how effective** are these surveillance and screening technologies in this country?

Probe: Could you tell me why you think so?

1. What evaluations are being done on **suitability** of the available technologies?

Probe: comparing accuracy, field-usage, cost, ease-of-use

1. Can you tell me if any others are doing surveillance and screening of medicines and health products?
2. Would you know if local manufacturers or others (importers, distributors) are using these detection technologies?

Probe: Which agencies/groups/NGOs/companies, pharmacists, physicians, MOH, National Lab staff, customs, port authority, law enforcement are testing medicines and other health products in this country?

**Action**:

1. When products are found to be SSFFC then, what actions are taken?
2. What happens with the medicine or health product quality testing results?

Probes: are they logged, report issued, who is report sent to, shared with public and private sector, procedure for reporting the results, any awareness raising for consumers/patients

Probe: enforcement action, recall of products, raids, arrests

1. Are the results shared with other countries or regional/global initiatives?
2. **Closing questions**

**We are almost at the end of the interview. I would like to close with a few general questions.**

1. What recommendations do you have for successful detection of SSFFC medicines and health products in _____(your country)?

Probe: Could you tell me why?

1. **What would you consider to be ideal qualities of the technology that you would like to use for surveillance and screening purposes in this country?**

Probes: cost, size, various functions, and kinds of information you can get from it, how it works to get that information

1. If there were information about surveillance and screening technologies that are being used, would you use the information?
2. How would you use this information if it were available to you?
3. Is there anything else that you’d like to share that we have not talked about?

Thank you very much for taking the time to participate in the interview and for sharing your thoughts and concerns. If you have any further questions feel free to contact us.

Ameena Nalim ([Fan7@cornell.edu](mailto:Fan7@cornell.edu))

Lukas Roth ([lmr@usp.org](mailto:lmr@usp.org))
