# Supplementary material for: Global landscape assessment of screening technologies for medicine quality assurance: stakeholder perceptions and practices from ten countries
Source: Global Health. 2018 Apr 25;14:43. doi: 10.1186/s12992-018-0360-y (PMC5922304; doi:10.1186/s12992-018-0360-y)
Supplement: Supplementary file 2 — Screening technologies by country, government Regulator [R], Manufacturer [M], and Distributor/Pharmacy [DP]. Identifies the screening technologies currently being used by the organizations interviewed, grouped by region and country. (DOCX 42 kb) [file 12992_2018_360_MOESM2_ESM.docx]

**Additional file 2. Screening technologies by country, government Regulator [R], Manufacturer [M], and Distributor/Pharmacy [DP].**

**Africa and the Americas**

| **Screening Technology** | **Argentina** | | | | | **Mexico** | | | | **Nigeria** | | | | | **USA** | | | | **Zimbabwe** | | | | |
| --- | --- | --- | --- | --- | --- | --- | --- | --- | --- | --- | --- | --- | --- | --- | --- | --- | --- | --- | --- | --- | --- | --- | --- |
|  | **R1** | **M1** | **M2** | **DP1** | **DP2** | **R1** | **M1** | **DP1** | **DP2** | **R1** | **M1** | **M2** | **DP1** | **DP2** | **R1** | **M1** | **M2** | **DP1** | **R1** | **M1** | **M2** | **DP1** | **DP2** |
| Documentation check  (inclusive of WHO checklist) | **✓** | **✓** | **✓** | **✓** | **✓** | **✓** | **✓** | **✓** | **✓** | **✓** | **✓** | **✓** | **✓** | **✓** | **✓** | **✓** | **✓** | **✓** | **✓** | **✓** | **✓** | **✓** | **✓** |
| Alternate Light Sources |  |  |  |  |  |  |  |  |  |  |  |  |  |  | **✓** |  |  |  |  |  |  |  |  |
| Handheld Refractometer |  | **✓** |  |  |  |  |  |  |  |  |  |  |  |  |  |  |  |  | **✓** |  |  |  |  |
| Handheld Viscometer |  | **✓** |  |  |  |  |  |  |  |  |  |  |  |  |  |  |  |  |  |  |  |  |  |
| Portable colorimetric tests |  | **✓** |  |  |  |  | **✓** |  |  |  |  |  |  |  |  |  |  |  |  |  |  |  |  |
| Simple disintegration |  |  |  |  |  |  |  |  |  | **✓** | **✓** |  |  |  |  |  |  |  | **✓** |  |  |  |  |
| Capillary electrophoresis |  |  |  |  |  |  |  |  |  |  |  |  |  |  |  |  |  |  |  |  |  |  |  |
| Portable conductometer |  | **✓** |  |  |  |  |  |  |  |  |  |  |  |  |  |  |  |  |  |  |  |  |  |
| Portable TLC |  |  |  |  |  |  |  |  |  | **✓** |  |  |  |  |  |  |  |  | **✓** |  |  |  |  |
| Portable GC-MS |  |  |  |  |  |  |  |  |  |  |  |  |  |  |  |  |  |  |  |  |  |  |  |
| Handheld IR |  |  |  |  |  |  |  |  |  |  |  |  |  |  |  |  |  |  |  |  |  |  |  |
| Handheld NIR |  |  | **✓** |  |  |  | **✓** |  |  |  |  |  |  |  | **✓** | **✓** | **✓** |  |  |  |  |  |  |
| Handheld FTIR |  |  |  |  |  |  |  |  |  |  |  |  |  |  | **✓** |  |  |  |  |  |  |  |  |
| Handheld UV-Vis |  |  |  |  |  |  |  |  |  |  |  |  |  |  |  |  |  |  |  |  |  |  |  |
| Handheld Raman |  | **✓** | **✓** |  |  |  | **✓** |  |  | **✓** |  |  |  |  | **✓** | **✓** | **✓** |  |  |  |  |  |  |
| Portable IMS |  |  |  |  |  |  |  |  |  |  |  |  |  |  | **✓** |  |  |  |  |  |  |  |  |
| Handheld NMR |  |  |  |  |  |  |  |  |  |  |  |  |  |  |  |  |  |  |  |  |  |  |  |
| Handheld NQR |  |  |  |  |  |  |  |  |  |  |  |  |  |  |  |  |  |  |  |  |  |  |  |
| Handheld XRD |  |  |  |  |  |  |  |  |  |  |  |  |  |  |  |  |  |  |  |  |  |  |  |
| Handheld XRF |  |  |  |  |  |  |  |  |  |  |  |  |  |  | **✓** |  |  |  |  |  |  |  |  |
| Portable MS |  |  |  |  |  |  |  |  |  |  |  |  |  |  |  |  |  |  |  |  |  |  |  |
| Portable sterility |  | **✓** |  |  |  |  | **✓** |  |  |  |  |  |  |  |  |  |  |  |  |  |  |  |  |

**S3 Annex continued. Screening technologies by government Regulator [R], Manufacturer [M], and Distributor/Pharmacy [DP].**

**Eastern Mediterranean, Asia, and Western Pacific**

| **Screening Technology** | **China** | | | | **Egypt** | | **India** | | | | | **Jordan** | | **Philippines** | | | | |
| --- | --- | --- | --- | --- | --- | --- | --- | --- | --- | --- | --- | --- | --- | --- | --- | --- | --- | --- |
|  | **R1** | **M1** | **M2** | **DP1** | **R1** | **M1** | **R1** | **M1** | **M2** | **DP1** | **DP2** | **R1** | **M1** | **R1** | **M1** | **M2** | **DP1** | **DP2** |
| Documentation check  (inclusive of WHO checklist) | **✓** | **✓** | **✓** | **✓** | **✓** | **✓** | **✓** | **✓** | **✓** | **✓** | **✓** | **✓** | **✓** | **✓** | **✓** | **✓** | **✓** | **✓** |
| Alternate Light Sources | **✓** |  |  |  |  |  |  |  |  |  |  |  |  |  |  |  |  |  |
| Handheld Refractometer |  |  |  |  |  |  | **✓** | **✓** | **✓** |  |  |  |  |  |  |  |  |  |
| Handheld Viscometer |  |  |  |  |  |  |  |  |  |  |  |  |  |  |  |  |  |  |
| Portable colorimetric tests | **✓** |  |  |  |  |  | **✓** | **✓** | **✓** |  |  |  |  | **✓** |  |  |  |  |
| Simple disintegration | **✓** |  |  |  |  |  | **✓** | **✓** | **✓** |  |  |  |  | **✓** |  |  |  |  |
| Capillary electrophoresis |  |  |  |  |  |  |  |  |  |  |  |  |  |  |  |  |  |  |
| Portable conductimeter |  |  |  |  |  |  |  |  |  |  |  |  |  |  |  |  |  |  |
| Portable TLC | **✓** |  |  |  |  |  | **✓** | **✓** | **✓** |  |  |  |  | **✓** |  |  |  |  |
| Portable GC-MS |  |  |  |  |  |  |  |  |  |  |  |  |  |  |  |  |  |  |
| Handheld IR |  |  |  |  |  |  |  | **✓** |  |  |  |  |  |  |  |  |  |  |
| Handheld NIR | **✓** |  |  |  |  |  | **✓** | **✓** | **✓** |  |  |  | **✓** |  |  |  |  |  |
| Handheld FTIR | **✓** |  |  |  |  |  |  |  |  |  |  |  |  |  |  |  |  |  |
| Handheld UV-Vis |  |  |  |  |  |  |  |  |  |  |  |  |  |  |  |  |  |  |
| Handheld Raman | **✓** | **✓** | **✓** |  |  |  | **✓** | **✓** | **✓** |  |  | **✓** | **✓** |  |  |  |  |  |
| Portable IMS |  |  |  |  |  |  |  |  |  |  |  |  |  |  |  |  |  |  |
| Handheld NMR |  |  |  |  |  |  |  |  |  |  |  |  |  |  |  |  |  |  |
| Handheld NQR |  |  |  |  |  |  |  |  |  |  |  |  |  |  |  |  |  |  |
| Handheld XRD |  |  |  |  |  |  |  | **✓** |  |  |  |  |  |  |  |  |  |  |
| Handheld XRF | **✓** |  |  |  |  |  | **✓** | **✓** |  |  |  |  |  |  |  |  |  |  |
| Portable MS |  |  |  |  |  |  |  |  |  |  |  |  |  |  |  |  |  |  |
| Portable sterility |  |  |  |  |  |  |  |  |  |  |  |  |  |  |  |  |  |  |
