# Supplementary material for: Global landscape assessment of screening technologies for medicine quality assurance: stakeholder perceptions and practices from ten countries
Source: Global Health. 2018 Apr 25;14:43. doi: 10.1186/s12992-018-0360-y (PMC5922304; doi:10.1186/s12992-018-0360-y)
Supplement: Supplementary file 3 — Additional interview information on use of screening technologies in countries surveyed. Highlights additional information gathered from interviews, which was not directly used in the results of discussion sections. (DOCX 27 kb) [file 12992_2018_360_MOESM3_ESM.docx]

**S4 Annex. Additional interview information on use of screening technologies in countries surveyed.**

Legend

No additional information not already included in results

| **Use of Screening Technologies in Countries Surveyed** | | | |
| --- | --- | --- | --- |
|  | **Regulators [R]** | **Manufacturers [M]** | **Distributors and Pharmacies [DP]** |
| **NIGERIA** | | | |
| **Quality Control (QC) Laboratory** | • Strong, well-equipped laboratories, some are ISO 17025 • Seven different specialized laboratories around the country | • Strong, well-equipped laboratory for testing finished products and raw materials | • No laboratory, testing is outsourced |
| **Challenges faced by QC Lab** | • Large numbers of samples to be tested  • High cost of equipment  • Acquiring reference materials  • Servicing of equipment | • High cost of equipment |  |
| **Screening Technology (ST)** | • Minilab™ used at ports by PID • Uses TruScan™ at borders, trade fairs, huge open markets, pharmacies, manufacturer's factories | • Not using any screening technologies | • Not using any screening technologies |
| **Reasons for using/not using ST** | • *"Conventional testing in laboratories is very, very expensive and time consuming"* | • Not familiar with the capabilities of STs | • Cannot check large numbers of products  • Purchase products from reputable manufacturers only  • Use barcodes on products  • Regulators use TruScan™ to spot check products on shelves |
| **Limitations of using ST** | • Cannot use it for all products • Power shortages affect the full use of equipment  • Updating software online is a major challenge  • Training of users requires funding. Trained personnel retire or transfer to other jobs. |  |  |
| **Ideal Qualities of ST** | • Affordable, easy to use, fast and accurate, requires less training, non-destructive of packaging or samples, touch-screen technology, with rechargeable battery and dual power source  • Software should be secure, and not crash | | |
| **ZIMBABWE** | | | |
| **Quality Control (QC) Laboratory** | • Strong, well-equipped WHO prequalified laboratory | • No QC laboratory • Raw material shipments checked at warehouses | • No laboratory |
| **Challenges faced by QC Lab** | • Lack of government funding for lab activities • High cost of reference standards and reagents • Lack of trained personnel for handling equipment and for sampling • Traveling distance (~600 km) between border check points and testing laboratory |  |  |
| **Screening Technology (ST)** | • Not using any screening technologies | • Not using any screening technologies | • Not using any portable/hand held screening technologies • Check barcodes, and registration number for identification of products |
| **Reasons for using/not using ST** | • Procuring TruScan™ to screen for SF using a library of spectra without destroying the sample | • Lack of knowledge about portable and handheld technologies | • High cost and time-consuming to check each product • Prefer regulators to do random checking of products on the shelf |
| **Limitations of using ST** |  |  |  |
| **Ideal Qualities of ST** | • Fast and accurate identification; comes with a spectral library for all products • Accurate product identification is a priority—using UV or IR, or NMR, “*as quantitative analyses would be in the lab*” • Improves the work flow and makes detection foolproof  • Automated checking for registration number instead of the visual checking • Knowledgeable about Raman technology like TruScan™, therefore preferred over others | | |
| **ARGENTINA** | | | |
| **Quality Control (QC) Laboratory** | • Strong, well-equipped laboratories | • Strong laboratories for testing starting materials and finished products  • One manufacturer has a laboratory especially for screening for falsified products using benchtop UV cameras, microscopes, and IR | • No laboratory • Monitor the temperature and relative humidity where medicines are stored  • Can request the certificate of analysis from ANMAT for suspect products |
| **Challenges faced by QC Lab** |  | • Difficult bringing new equipment into Argentina because of strict government legislation and policies • High cost of equipment |  |
| **Screening Technology (ST)** | • Not using any screening technologies | • Use handheld Raman and NIR spectrometers | • Not using any screening technologies |
| **Reasons for using/not using ST** | • Use HPLC and IR for identification of drugs | • *"It is a piece of technology that saves a lot of time and obviously a lot of reagents. It also prevents contamination because we don't have to open one drum after the next."*  • Use NIR because “*it was the most sustainable technology on the market and it is fast*” • Transitioning from one tool to another is time-intensive since libraries are already constructed | • Don't believe there is a need for any STs • Use National Traceability System as tool for assuring quality and product origin • Monitor temperatures carefully where cold storage of medicines is required |
| **Limitations of using ST** |  | • No particular challenges in using Raman • Only qualified quality control chemists use the Raman  • Lack of knowledge about other new technologies available  • High cost and procurement are issues as there are no in-country agents |  |
| **Ideal Qualities of ST** |  | | |
| **MEXICO** | | | |
| **Quality Control (QC) Laboratory** | • National testing laboratory tests random samples of raw materials and finished products taken by regulatory inspectors | • Use various analytical techniques for testing raw materials received | • No laboratory |
| **Challenges faced by QC Lab** | • Lack of access to methodologies for analysis of new products  • Use external private laboratories to cope with the large volume of samples |  |  |
| **Screening Technology (ST)** | • Not using any screening technologies • Inspectors perform visual inspection of packaging for safety seal, name, batch number, and expiry date | • Some manufacturers beginning to evaluate NIR and Raman for use | • Not using any screening technologies • Perform visual inspection of packages to check intact seals, batch number, and product appearance |
| **Reasons for using/not using ST** | • STs not recognized as standard by the national pharmacopeia | • Law 059 requires manufacturers to identify raw materials, excipients, and API by sampling all containers | *• "A technology that would enable us to guarantee that the product is good [by testing the drug] in addition to visual inspection would be good. However, destructive testing of high price drugs would be too costly."* |
| **Limitations of using ST** |  | • NIR cannot detect certain inorganic compounds or certain functional groups |  |
| **Ideal Qualities of ST** | • Identification of all materials/substances of interest • Ease of sampling (need for a special area in warehouse for sampling, need to open containers) • Speed of results acquisition • Memory capacity of barcode reader and built-in-camera • Easy method development | | |
| **USA** | | | |
| **Quality Control (QC) Laboratory** | • Strong, well-equipped laboratories | • Strong, well-equipped laboratories | • No laboratory |
| **Challenges faced by QC Lab** |  |  |  |
| **Screening Technology (ST)** | • Use visual examination, alternate light sources (ALS), handheld Raman, FT-IR, XRF, and IMS technologies | • Handheld Raman NIR are used by one manufacturer for testing suspect products recovered from the grey market  • Another manufacturer uses Raman for post marketing quality surveillance and thinks *“over 90% of manufacturers likely using portable Raman TruScan™”* | • Not using any screening technologies |
| **Reasons for using/not using ST** | • Technologies allow for the screening of drug products entering the U.S.  • Inclusion of both product and packaging in the device libraries allows rapid evaluation at points of entry • The commercial devices are expensive compared to the in-house developed ALS device | • Instrument vendors provide a lot of training and support • Can distinguish organic substances very well | • Would not need to use screening technologies as distributors use a track and trace system  • Use only large distributors and major wholesalers and stay away from purchasing stocks from smaller, secondary suppliers. |
| **Limitations of using ST** | • Updating and maintaining libraries as approved products are added to the marketplace, as new adulterants are discovered, or as packaging formats change  • Keeping technology current, ensuring new staff are trained, and ensuring the technologies are used on a regular/routine basis | • Handheld Raman cannot reliably authenticate all company medicines; results depend on the complexity of the chemical composition |  |
| **Ideal Qualities of ST** | • *"Handheld Raman devices are expensive but effective”*  • *"An ideal authentication technology would have big data capabilities to permit analysis and linkage of samples tested."* | | |
| **EGYPT** | | | |
| **Quality Control (QC) Laboratory** | • Strong, well-equipped network of laboratories run by regulatory authority | • Strong, well-equipped laboratory |  |
| **Challenges faced by QC Lab** |  | • Sometimes reference standards and other materials received do not comply to claimed standards |  |
| **Screening Technology (ST)** | • Not using any screening technologies • Perform visual inspection of packages | • Not using any screening technologies | • Not using any screening technologies • Some pharmacies use barcoding, but not all |
| **Reasons for using/not using ST** | • Regulatory inspectors have not proposed the use of STs • Less screening at ports of entry as most products are made in country | • STs can be used to screen incoming materials |  |
| **Limitations of using ST** |  |  |  |
| **Ideal Qualities of ST** | • *"Having such technologies in different countries, in Africa, in Middle East, Far East, will benefit us. Even if we do not get access to it in Egypt, but its presence in other countries will be a benefit to us."* | | |
| **JORDAN** | | | |
| **Quality Control (QC) Laboratory** | • Strong, well-equipped laboratory for testing • Laboratory runs tests on all products (imported and local) prior to registration, as well as suspect SF | • Multiple, regional QC labs equipped with HPLC, UVLC for finished product analysis |  |
| **Challenges faced by QC Lab** | • *"We don't have enough analysts to test all products”*  • Insufficient equipment - *“we have to cover all the tests that we have to run…we can't buy every year many…types of instruments…"* | • *"Maybe would like to use Raman for finished product testing at the lab, if it will be fast and reduce time while giving a quantitative result"* |  |
| **Screening Technology (ST)** | • Not currently using any STs, but have in the past | • Use Raman TruScan™ and/or NIR microPHAZIR™ RX Analyzer for identification of raw materials only |  |
| **Reasons for using/not using ST** | • Insufficient library to scan all products  • ST results will not be accepted by the authorities to prove a product as SF | • Affordable, non-destructive, safe for the user  • NIR was not as expensive as Raman or NMR |  |
| **Limitations of using ST** | • Cross contamination between the first tablet and the next tablet to be read; needs cleaning between samples | • Prefer Raman as more accurate but takes more time, about 9.0 sec/sample  • NIR takes about 3.0 sec/sample, faster identification of raw material sample  • Creating spectral library is a challenge |  |
| **Ideal Qualities of ST** | • Smaller than existing technologies with a touchscreen • Improve issue of cross-contamination between the first and second tablet | | |
| **INDIA** | | | |
| **Quality Control (QC) Laboratory** | • Strong, well-equipped laboratory | • Well-equipped HQ laboratory for research with several smaller laboratories at different sites for testing |  |
| **Challenges faced by QC Lab** |  |  |  |
| **Screening Technology (ST)** | • Use handheld Raman, NIR, and XRF spectrometers | • A MASS code is placed by manufacturers on the product for track and trace • Track and trace used in parts of India: expensive and difficult to implement by smaller companies | • Not using any screening technologies • Check barcodes and perform visual inspection |
| **Reasons for using/not using ST** | • Excellent capacity at QC labs | • Excellent capacity at QC labs • After developing the models, handheld ST are fast and easy to use with increasing amounts of data | • Barcodes, tablet codes, and tamper resistant seals on products as well as batch numbers are checked |
| **Limitations of using ST** | • NIR spectroscopy cannot test coated (film and enteric) solid dosage forms | • Time-consuming preparation of models and methods • Building a spectral library and regularly updating it  • Different companies cannot share spectral libraries as spectra are specific for each product formulation • Only spectra of raw materials with the same particle size may be shared |  |
| **Ideal Qualities of ST** | • Track and trace systems are ideal - a barcode that is generated using a random number generator, for anyone to track the product from manufacture to consumer • *"[Track and trace] Is a better solution because the patient can easily identify, nobody… can able to reproduce; Harmonization of these barcodes between countries is important.”* | | |
| **PHILIPPINES** | | | |
| **Quality Control (QC) Laboratory** | • Three fairly functional, well-equipped 3 FDA laboratories  • Nine total labs (government and private) for testing food products, not pharmaceuticals | • Visual check of packaging for identifying SF and some use of HPLC, TLC, GC | • No laboratory |
| **Challenges faced by QC Lab** |  |  |  |
| **Screening Technology (ST)** | • Six sentinel FDA labs use 9 Minilabs™ to screen anti-TB medicines | • Not using any screening technologies | • Not using any screening technologies  • Perform visual inspection of packaging |
| **Reasons for using/not using ST** | • High cost and need to train users • When FDA was under DOH, providing inspectors with ST was not previously their work | • High cost of IR prevents use on finished product line and incoming raw materials  • Lack of knowledge about hand held STs  • Looking at acquiring a handheld GC analyzer | • If there are complaints, the FDA does analysis of the medicines |
| **Limitations of using ST** | • Training Minilab™ users • Managing supplies needed until next purchase or delivery  • Replacing reagents needs special permits |  |  |
| **Ideal Qualities of ST** | • Good follow up maintenance and customer service  • User training provided  • *"If we could translate data gathered from these handheld instruments to our PCs directly without requiring any special programs to read and generate quick reports"* | | |
| **CHINA** | | | |
| **Quality Control (QC) Laboratory** | • CFDA and the NIFDC have QC laboratories in multiple locations • QC labs per province depend on size of the province • Standard laboratory equipment used in most provinces | • HPLC and IR are used to check counterfeit medicines for identification of the API |  |
| **Challenges faced by QC Lab** | • Using high resolution powerful devices that read spectral peaks for the drugs as well as for impurities such as salts in the mobile phase.  • Using columns and developing the spectra for mass spectroscopy (MS) is a challenge |  |  |
| **Screening Technology (ST)** | • Use of Raman and NIR STs varies depending on the province in China • Some larger provinces (of 31) use STs in mobile van testing laboratories | • Some manufacturers use instruments like TruScan™ for internal use to check raw materials • Perform visual inspection, and check barcodes | • Not using any screening technologies |
| **Reasons for using/not using ST** | • Many provincial governments can't afford STs  • Mobile labs include benchtop devices and that can test food and drug samples from the market  • Can't make a final decision based results from STs; lab-based equipment is still needed • STs will not be useful in customs and ports | *"Current monograph does not have guidelines for using STs as part of release testing"* | *"We are not capable for such tests in terms of facility, equipment and technology."* |
| **Limitations of using ST** | • Suspicious samples are brought back to the laboratory for testing • NIR and Raman are good for screening but not for confirmation |  |  |
| **Ideal Qualities of ST** | • Handheld Raman and NIR spectrophotometers considered ideal as agents are available in-country for service and repair | | |
